# Cholecystostomy for Gall-Stones, with Occlusion of the Cystic Duct

**Published:** 1893-11

**Authors:** 


### Editorial.

### CHOLECYSTOSTOMY FOR GALL-STONES, WITH OCCLUSION OF THE CYSTIC DUCT.

In a previous notice of the procedure indicated for relief in occlusion of the common bile duct, when the cystic duct is permeable, anastomosis with the intestinal canal was urged. To meet the case of occlusion of the cystic duct, when the common duct is open and conveys the bile into the duodenum quite a different process is requisite. I had occasion to operate before the students of the Southern Medical College in a case recently, presenting the latter conditions in the person of a woman 50 years old, who had suffered in former years with hepatic colic and jaundice. She had, however, been free from these attacks for more than a year; and an enlargement of the gall-bladder was recognized below the margin of the liver, leading to the diagnosis of obstruction with retained gall-stones.

An incision, three inches in length, diagonally below the points of the ribs on the right side, revealed the distended gall-bladder. This being secured by a strong silk ligature, passed through the wall with a curved needle, a free opening with a bistoury was made in the fundus, which was drawn out of the external incision so as to give exit to the collection of mucus. Passing the index finger on the outside of the lower wall of the gall-bladder and a calculus scoop within the cavity of the sac, six faceted biliary calculi were removed from the gall-bladder.

The gall-bladder was anchored to the parietes by carrying the ligature already passed through it, out through the sides of the external incision and knotting the ends so as to close the wound at that point. Other interrupted sutures were used to approximate the upper part of the opening. A large drainage tube was inserted into the cavity of the gall-bladder and brought out at the lower angle of the incision, after having stitched the serous surface of the opening into the sac to the peritoneal lining of the parietes. The wound was dressed subsequently with iodoform, and iodoform gauze, covered with a layer of absorbent cotton, secured by a roller bandage around the body.

The retention suture was removed on the third day and the interrupted sutures on the eighth day.

The temperature did not at any time exceed 100 F., and the drainage tube was gradually cut off and finally removed on the twelfth day. There was considerable discharge of watery mucus for the first week, but this gradually ceased and on the nineteenth day there was no longer any discharge. The wound had all closed excepting at the lower end, where a piece of lint has been kept to prevent union of the skin. There has been no trace of bile in the discharge at any time, and it is clear that the gall bladder will become atrophied, and ultimately obliterated, leaving the bile to take its course through the common duct directly into the duodenum. The patient left for her home near Austell on the twenty-second day, with the wound healed and free from trouble of any kind. J. McF. G.

